# Epigenetics in the formation of pathological aggregates in amyotrophic lateral sclerosis

**DOI:** 10.3389/fnmol.2024.1417961

**Published:** 2024-09-03

**Authors:** Veronica Noches, Danae Campos-Melo, Cristian A. Droppelmann, Michael J. Strong

**Affiliations:** ^1^Molecular Medicine Group, Robarts Research Institute, Schulich School of Medicine and Dentistry, Western University, London, ON, Canada; ^2^Department of Clinical Neurological Sciences, Schulich School of Medicine and Dentistry, Western University, London, ON, Canada

**Keywords:** ALS, epigenetics, protein aggregates, DNA methylation, histone modifications, chromatin remodeling enzymes, non-coding RNAs, RNA modifications

## Abstract

The progressive degeneration of motor neurons in amyotrophic lateral sclerosis (ALS) is accompanied by the formation of a broad array of cytoplasmic and nuclear neuronal inclusions (protein aggregates) largely containing RNA-binding proteins such as TAR DNA-binding protein 43 (TDP-43) or fused in sarcoma/translocated in liposarcoma (FUS/TLS). This process is driven by a liquid-to-solid phase separation generally from proteins in membrane-less organelles giving rise to pathological biomolecular condensates. The formation of these protein aggregates suggests a fundamental alteration in the mRNA expression or the levels of the proteins involved. Considering the role of the epigenome in gene expression, alterations in DNA methylation, histone modifications, chromatin remodeling, non-coding RNAs, and RNA modifications become highly relevant to understanding how this pathological process takes effect. In this review, we explore the evidence that links epigenetic mechanisms with the formation of protein aggregates in ALS. We propose that a greater understanding of the role of the epigenome and how this inter-relates with the formation of pathological LLPS in ALS will provide an attractive therapeutic target.

## Introduction

Amyotrophic lateral sclerosis (ALS) is characterized by the relentless degeneration of motor neurons subserving both upper and lower motor neuron functions (UMN & LMN, respectively), ultimately giving rise to progressive muscle weakness, spasticity, and atrophy, with lethal respiratory failure within two to five years of the disease onset (Strong et al., 2005; Robberecht and Philips, 2013). The majority of ALS cases are sporadic (sALS) with the remainder being genetic, often in association with family history (fALS). Approximately two out of three individuals with fALS and one out of ten individuals with sALS have a mutation in at least one of the over forty genes associated with the condition. These genes vary in the degree of evidence supporting their association with ALS, with many having limited or weak evidence (http://alsod.iop.kcl.ac.uk) (Ghasemi and Brown, 2018; Masrori and Van Damme, 2020).

While classically ALS is considered to be a pure disorder of the motor neurons, it is now accepted that over 50% of ALS patients have an associated disorder of frontotemporal dysfunction, including either presenting with or developing frontotemporal dementia (FTD) (Hardiman et al., 2017; Strong et al., 2017). The progressive degeneration of motor neurons in ALS is accompanied by the formation of a broad array of nuclear and cytoplasmic inclusions which are driven by liquid-to-solid phase separation (Chakraborty and Zweckstetter, 2023; Shen et al., 2023). Amongst a broad array of proteins associated with these inclusions are RNA-binding proteins (RBPs), of which inclusions containing the TAR DNA-binding protein 43 (TDP-43) are observed in the vast majority of degenerating neurons (Ling et al., 2013).

Considering the complexity of this disease at the molecular level (Goutman et al., 2022), the mechanisms associated with the pathogenesis of ALS are currently a priority area of study. In recent years, it has been suggested that epigenetic mechanisms could contribute to the etiology of ALS (Bennett et al., 2019). A contemporary definition of epigenetics by Cavalli and Heard indicates that it is “the study of molecules and mechanisms that can perpetuate alternative gene activity states in the context of the same DNA sequence” (Cavalli and Heard, 2019). This includes both mitotic and transgenerational inheritance, as well as the long-term maintenance of gene activity or chromatin states in the absence of cell division, with a DNA sequence that is biological system-dependent (Cavalli and Heard, 2019). Nowadays, the consensus is that epigenetics includes DNA methylation, histone modifications, and RNA-based mechanisms, a multilayer process that controls gene expression through altering DNA packing and accessibility and regulates the levels of proteins using RNA machinery (Dulac, 2010; Gibney and Nolan, 2010; Aristizabal et al., 2020; Jiang et al., 2023). The most strongly ALS-associated genes for which there is evidence of a potential association between aggregate formation and alterations in the epigenome are included in Table 1.

**Table 1:** Genes associated to ALS, NCIs formation and epigenetic regulations.

Gene	Inclusions formation	Direct or indirect epigenetic regulation
*SOD1*	High propensity to misfold and form abnormal aggregates (Ayers et al., 2017).	Hypermethylation in blood DNA; demethylation in gene promoter (Oates and Pamphlett, 2007; Coppede et al., 2018); miR-155 (Bi et al., 2022a), miR-206 (Zhang et al., 2015), lncRNA SOD1-DT (Guerra et al., 2023).
*NEFH*	Hyaline conglomerate inclusion (Xiao et al., 2006).	MiR-9-5p, miR-20b-5p, miR-92a-3p, and miR-223-3p (Campos-Melo et al., 2018).
*ANG*	Alter the formation of stress granules (Thiyagarajan et al., 2012).	Promoter methylation and histone modification (Sheng et al., 2014); miR-182-5p (Li et al., 2022a), miR-409-3p (Weng et al., 2012).
*CHMP2B*	Accumulation of SQSTM1/p62 and ubiquitin-positive inclusions (Ghazi-Noori et al., 2012).	Knows as chromatin modifying protein 2B, with epigenetic contribution to FTD (Veerappan et al., 2013).
*TARDBP*	Ubiquitin-positive inclusions (Arai et al., 2006; Neumann et al., 2006).	Decrease in methylated DNA (Appleby-Mallinder et al., 2021); Interaction with CHD2 (Berson et al., 2017); miR-194 and miR-b2122 (Hawley et al., 2017), miR-132-3p and miR-132-5p (Kawahara and Mieda-Sato, 2012), miR-183-5p (Kim et al., 2023), miR-27b-3p and miR-181c-5p (Campos-Melo et al., 2013; Hawley et al., 2020).
*FUS/TLS*	Basophilic inclusions (Suzuki et al., 2012; Tibshirani et al., 2015); Ubiquitinated/TDP-43–positive NCIs are granular, vermiform, and skein-like inclusions as well as Bunina bodies (Hewitt et al., 2010).	Decrease of H4R3me2; reduced levels of H3S10ph, and H2BT129ph; and hypoacetylation of H3K14ac and 56 H3K56ac (Chen et al., 2018); miR-194 and miR-b2122 (Hawley et al., 2017), miR-141(Svetoni et al., 2017), miRNA-200a-3p (Chang et al., 2020a), miR-133a-5p (Zheng et al., 2020), miR-219a-2-3p (Yang et al., 2020), miR-378 (Ma et al., 2014), miR-141 and miR-200ª (Dini Modigliani et al., 2014), miR-378 (Lee et al., 2007), lncRNA XIST and miR-200a (Zhu et al., 2018), circRNA-0004904 (Dai and Liu, 2021), circRNA_0000285 (Chen et al., 2019).
*ARHGEF28*	Neurofilament aggregate micronuclei of TDP-43 co-aggregate with RGNEF under metabolic stress (Droppelmann et al., 2013; Droppelmann et al., 2019).	miR-194 and miR-b2122 (Hawley et al., 2017).
*VCP*	SQSTM1/p62 -positive, ubiquitin-positive, and TDP–43–positive inclusions (Koppers et al., 2012; Buchan et al., 2013).	Ubiquitinated substrates on chromatin (Vaz et al., 2013). miR-129-5p (Liu et al., 2012), miR-339-5p (Bi et al., 2022b).
*OPTN*	Ubiquitin and TDP-43 -positive skein-like and round hyaline inclusions (Maruyama et al., 2010; Hortobagyi et al., 2011).	Methylation modification (Korac et al., 2013; Chen et al., 2022a; Shu et al., 2023). miR-331-3p and miR-9-5p (Chen et al., 2021a), miR-106b-93-25 cluster (Zhang et al., 2021).
*ATXN2*	Filamentous TDP-43 inclusions (Hart et al., 2012).	The CpG of gene promoter can be methylated (Laffita-Mesa et al., 2012). miR-873-3p and LINC00941 (Fang et al., 2021).
*C9orf72*	Cytoplasmic and nuclear granular inclusions (Tada et al., 2018)	The hypermethylation of the CpG island (Xi et al., 2015); methylation of promoter (Bauer, 2016).
*SQSTM1*	Colocalizes with FUS/TLS and TDP-43 in ubiquitinated inclusions (Deng et al., 2010).	DNA methylation in the promoter (Lee et al., 2021); histone and chromatin ubiquitination (Wang et al., 2016b). miR-183-5p (Kim et al., 2023), miR-331-3p and miR-9-5p (Chen et al., 2021a), miR-181a (Goljanek-Whysall et al., 2020), miRNA −93 (Huang et al., 2019), miR-17-5p (Li et al., 2018), miR-372 (Yeh et al., 2015), miR-17, miR-20, miR-93, miR-106 (Meenhuis et al., 2011), miR-361 (You et al., 2022), miR-7-5p (Liu et al., 2022b), miR-361-5p (Zeng et al., 2021), miR-145 (Higashi et al., 2015), lnc-MEG3 (Zhou et al., 2022a).
*UBQLN2*	Skein-like inclusions interact with TDP-43 into insoluble aggregates (Picher-Martel et al., 2015).	miR-155 (Yadav et al., 2017).
*TAF15*	Cytoplasmic TAF15-positive punctae (negative for TDP-43) (Couthouis et al., 2011; Mackenzie and Neumann, 2012).	miR-182-5p (Zhang et al., 2020).
*EWSR1*	Intrinsically aggregation–prone (Couthouis et al., 2012)	EWSR1 indirectly regulates the expression of microRNAs via induction of DROSHA (Kim et al., 2014). miR-141 (Svetoni et al., 2017).
*PFN1*	Ubiquitinated, insoluble aggregates that in many cases contain TDP-43 (Wu et al., 2012).	miR-19a-3p (Wang et al., 2018), miR-342-5p (Yu et al., 2023), miR-328-3p and LINC00963 (Yang et al., 2022), miR-1226-3p (Jian and Xia, 2021), miR-19a-3p (Wang et al., 2019), miR-182 (Liu et al., 2013).
*hnRNPA1 and hnRNPA2B1*	Skein-like inclusions, TDP-43 positive (Honda et al., 2015).	Dysregulated in ALS patient, related with downregulation of miRNA (Liguori et al., 2018). hnRNPA1: miR-206 (Fu et al., 2020), miR-339 (Chen et al., 2020), miR-15a-5p and miR-25-3p (Rodriguez-Aguayo et al., 2017), miR-490 (Zhou et al., 2016), miR-18a (Fujiya et al., 2014), miR-128 (Fung et al., 2019), lncRNA MIR4435-2HG (Li et al., 2023), linc02231 (Xu et al., 2023), lncRNA ANCR (Wen et al., 2020). hnRNPA2B1: miR-30c-5p (Wu et al., 2023), miR-326 (Luo et al., 2021), miR-146b-5p (Zhang and Li, 2019), miR-369 (Konno et al., 2015), lncRNA SOX2-OT (Zhang and Li, 2019).
*CHCHD10*	Accumulation and insolubility of TDP-43 in the cytoplasm (Woo et al., 2017; Baek et al., 2021).	Dysregulated in ALS patient, related with downregulation of miRNA (Liguori et al., 2018)
*MATR3*	Positive inclusions within the nuclei and cytoplasm (Johnson et al., 2014; Tada et al., 2018).	Dysregulated in ALS patient, related with downregulation of miRNA (Liguori et al., 2018)
*TUBA4A*	Aggregation with tubulin-binding proteins (Smith et al., 2014).	miRNA-1825 (Helferich et al., 2018).
*TBK1*	TDP-43 positive and SQSTM1/p62 positive inclusions in mor neurons (Oakes et al., 2017)	miR-199a (Wang et al., 2018), miR-200b-3p (Fang et al., 2023), MiR-217 (Salifu et al., 2023), miR-19a-3p, MiR-19a (Yin et al., 2019; Ni et al., 2022), miR-203 (Liu and Feng, 2015), miR-429 (Song et al., 2015), miR-15b (Chang et al., 2020b), miR-155-5p (Zhao et al., 2019).

These mechanisms mediate the diversified gene expression profiles in a variety of cells and tissues in multicellular organisms. Studying how these mechanisms can be perturbed in neurodegeneration, and most specifically are related to pathological biomolecular condensate formation in ALS, is a critical step in understanding the etiology of the disease beyond those causes mediated by heritable protein-coding gene mutations (Sultan and Day, 2011; Hwang et al., 2017).

### Formation of neuronal inclusions in ALS

Liquid–liquid phase separation (LLPS) segregates proteins and nucleic acids into liquid-like structures characterized as membrane-less organelles (MLOs). These structures play a crucial role in a variety of normal biological processes, such as chromatin organization, genomic stability, DNA damage response and repair, transcription, and signal transduction, and contribute to intracellular spatiotemporal coordination (Hyman et al., 2014; Nesterov et al., 2021; Wang et al., 2021a; Chen et al., 2022b). Additionally, LLPS is critical for the control of cellular functions such as metabolic processing and cellular compartment control (Hyman et al., 2014).

Nuclear bodies (NBs) are MLOs with various nuclear functions. The nucleolus is involved in pre-rRNA transcription, processing, and ribosomal ribonucleoprotein (RNP) assembly; nuclear speckles are associated with storage and assembly of spliceosomal components; nuclear stress bodies regulate transcription and splicing under stress; paraspeckles modulate gene expression by sequestering specific mRNAs and proteins; Promyelocytic leukemia gene product (PML) play key roles in genome stability, DNA repair, control of transcription, and viral defense; and Cajal bodies are involved in the biogenesis, maturation, and recycling of small RNAs (Lamond and Spector, 2003; Handwerger and Gall, 2006; Boulon et al., 2010).

Cytoplasmic MLOs, generally known as RNP granules, are essential in mRNA metabolism and homeostasis. These molecular structures include processing bodies (P-bodies) involved in post-transcriptional gene regulation and mRNA metabolism; stress granules (SGs) that play crucial roles in regulating mRNA metabolism, translation, and stress response pathways; and germ granules that orchestrate RNA regulation, genome integrity, epigenetic regulation, and stress responses in germ cells (Kedersha et al., 2005; Buchan, 2014; Jain et al., 2016; Luo et al., 2018).

In general, the interaction of the intrinsically disordered regions (IDRs) of proteins are responsible for their phase separation. IDRs are usually made up of a limited number of amino acids and/or repetitive sequence elements, referred to as low-complexity domains (LCDs) (Pakravan et al., 2021). Many ALS-related proteins have LCDs, making them prone to forming aggregates through LLPS. In ALS, dysfunctional LLPS can lead to pathological protein aggregation (liquid-to-solid phase transition), contributing to disease progression (Pakravan et al., 2021). As a result, to avoid aggregate formation and protein accumulation, both LLPS and clearance functions must be balanced properly.

In healthy cells, TDP-43 forms various oligomeric states within the nucleus through interactions of its N-terminal domains (Afroz et al., 2017). Under pathological conditions such as observed in ALS, TDP-43 becomes predominantly cytosolic with relative nuclear clearance and forms biomolecular condensates through LLPS. Both its N-terminal and C-terminal domains contribute to aggregation. In solution, LCDs of TDP-43 can self-aggregate with higher concentrations leading to the formation of gel-like structures. ALS-linked mutations in TDP-43 decrease its liquid properties and disrupt its phase-separation behavior (Conicella et al., 2016). Similar to TDP-43, mutations in the LCD of fused in sarcoma/translocated in liposarcoma (FUS/TLS) disrupt the phase-separating properties of the protein (Han et al., 2012). Full-length FUS/TLS has been observed to form droplets *in vitro*, affecting its LLPS by the RNA-to-protein ratio (Maharana et al., 2018).

In pathological states, MLOs can act as sites for the accumulation of abnormal protein species, contributing to the formation of neuronal inclusions in ALS or frontotemporal dementia (FTD) (Butti and Patten, 2018; Ismail et al., 2021). Neuronal inclusions are common in a wide range of neurodegenerative diseases (Dugger and Dickson, 2017; Wilson 3rd et al., 2023); however, there remains considerable debate as to whether the presence of cellular inclusions is beneficial or toxic for the cell (Lippi and Krisko, 2023). Liquid-to-solid phase transition in pathological conditions has been observed for several RBPs, including the ALS-related proteins TDP-43 (encoded by *TARDBP*) and FUS/TLS (encoded by *FUS*) (Carey and Guo, 2022), in a process that can be accelerated by ALS-associated mutations (Patel et al., 2015). These biomolecular condensates can be observed either in the nucleus or the cytoplasm of neurons (Banani et al., 2017).

#### Nuclear inclusions

Growing evidence points to the importance of structural and functional nuclear alterations in the etiology of many neurodegenerative illnesses. The formation of neuronal intranuclear inclusions (NIIs) is a consequence of sequestering essential nuclear factors by mutant proteins or RNAs within the nucleus (Woulfe, 2007). NIIs can trigger a progressive neurodegenerative condition typified by the presence of pathologic eosinophilic hyaline intranuclear inclusions. These inclusions are observed in both the central and peripheral nervous systems as well as in multiple visceral organs (Seilhean et al., 2004; Liu et al., 2022c; Tai et al., 2023). The presence of NIIs and the malfunction of the ubiquitin-proteasome system (UPS) such as elevated levels of ubiquitinated proteins and P62 protein are common pathological traits observed in neuronal intranuclear inclusion disease (NIID) as well as other neurodegenerative conditions. NBs, such as PML, function as sites for protein degradation and are linked to the ubiquitin-proteasome pathway (Mattsson et al., 2001). Intranuclear eosinophilic inclusions displaying ubiquitin, PML gene product, proteasome, and ataxin-3 immunoreactivity have been observed within the hippocampus and motor cortex in ALS (Kakita et al., 1997; Seilhean et al., 2004). TDP-43(+) neuronal inclusions with a distribution and morphology similar to NII and neuronal cytoplasmic inclusions (NCIs) have been observed in brain samples from patients with ALS and a concomitant FTD (Cairns et al., 2007; Seelaar et al., 2007).

#### Cytoplasmic inclusions

NCIs within degenerating motor neurons in association with UMN and LMN loss have long been the neuropathological hallmark of ALS. Classically, such inclusions were characterized based on their morphological appearance and included Bunina bodies which are small, electron dense, eosinophilic cytoplasmic ubiquitin-negative, TDP-43 positive inclusions (Tomonaga et al., 1978; Okamoto et al., 2008; Mori et al., 2014), Lewy body-like hyaline or skein-like inclusions which are ubiquitinated (Leigh et al., 1988; Lowe et al., 1988; Leigh et al., 1991; Okamoto et al., 2011); and neurofilament-rich “hyaline conglomerate inclusions” in degenerating motor neurons (Wada et al., 1999; Strong et al., 2005). The contemporary view of ALS, however, includes not only inclusions consisting of neuronal intermediate filaments but also the far more commonly observed cytoplasmic inclusions containing an array of proteins associated with RNA metabolism (Chou et al., 2018; Alberti and Dormann, 2019; Elbaum-Garfinkle, 2019). Amongst these, neuronal and glial cytoplasmic inclusions immunoreactive for TDP-43 are observed in virtually (approaching 97%) all ALS patients, except superoxide dismutase 1 (SOD1) and FUS/TLS cases (Andersen et al., 1995; Al-Sarraj et al., 2011; Boxer et al., 2011; Couthouis et al., 2011; Deng et al., 2011; Hortobagyi et al., 2011; Couthouis et al., 2012).

Little is known about the effect of NCIs directly on gene expression, but their presence can interfere with a broad range of normal cellular processes, including mRNA metabolism, protein trafficking, synaptic function, and intracellular signaling. Impaired expression of RBPs and their resulting aggregation lead to disruptions in RNA regulation, which manifest as abnormalities in processes such as splicing, polyadenylation, transport, translation, and decay of RNA targets (Kanai et al., 2004; Lagier-Tourenne et al., 2010; Nussbacher et al., 2019). Aggregates can have a broad variety of detrimental consequences in the cell, including interference with protein degradation pathways, disruption of cellular membranes, induction of oxidative stress, and activation of inflammatory responses. These toxic effects can ultimately lead to cellular dysfunction and, in severe cases, cell death (Stefanis, 2012; Prasad et al., 2019; Michalska and Leon, 2020).

### Epigenetic mechanisms involved in aggregate formation in ALS

While genetic mutations are well-established contributors to ALS pathology, emerging evidence suggests that epigenetic alterations also significantly influence aggregate formation in the disease. DNA methylation, histone modifications, and non-coding RNA-mediated mechanisms are among the key epigenetic processes implicated in ALS aggregate formation.

#### DNA methylation

One of the most studied epigenetic processes is DNA methylation in which a covalent transfer of a methyl group from S-adenosyl methionine (SAM) to carbon-5 of the cytosine pyrimidine ring of DNA occurs to form 5-methylcytosine (5mC). This modification in the DNA effectively inhibits gene transcription when adjacent to promoter regions (Robertson, 2005; Moore et al., 2013). DNA methylation is regulated by a family of DNA methyltransferases (DNMT) and is implicated in the regulation of several cellular processes (Bestor, 2000). Conversely, DNA demethylation is promoted by the ten-eleven translocation family of proteins (TET proteins), which oxidizes 5-methylcytosine (5-mC) to 5-hydroxymethylcytosine (5-hmC) (Guo et al., 2013). DNA methylation occurs almost exclusively on a cytosine followed by a guanine nucleotide (CpG dinucleotide). Over 80% of CpG dinucleotides located along the gene are typically subject to methylation; in contrast, CpG islands, regions that are greater than 500 base pairs in size with high CpG content, are hypomethylated (Bird et al., 1985; Esteller, 2005). This pattern changes with age such that CpG islands become hypermethylated and silenced (Robertson, 2005), whereas non-CpG sites are hypomethylated (Egger et al., 2004; Fraga et al., 2007).

Methylation of CpG (mCpG) has a role in transcription and replication by decreasing the speed of RNA/DNA polymerase and DNA helicase while stabilizing the DNA helix and raising its melting temperature *in vitro* (Rausch et al., 2021). Transcription factor (TF) binding is either facilitated or prevented when DNA methylation occurs at promoters of CpG islands, leading to gene silencing (Kass et al., 1997). TFs that recognize mCpG are classified as methyl-CpG binding proteins (MBPs) (Fournier et al., 2012; Shimbo and Wade, 2016) and are associated with increased chromatin density through LLPS. Specifically, methyl-CpG-binding protein 2 (MeCP2) induces compaction and LLPS of nucleosomal arrays *in vitro* and further enhances the formation of chromatin condensates by DNA methylation (Wang et al., 2020).

This is of importance given the evidence that DNA methylation is altered in ALS. It has been observed that the level of DNA methylation in the blood DNA of ALS patients with SOD1 mutation is notably higher compared to asymptomatic carriers or family members without SOD1 mutations. Also, a direct relationship between overall DNA methylation levels and the duration of the disease has been found in patients with SOD1 mutations (Coppede et al., 2018). An examination of DNA methylation across the entire genome in the frontal cortex of individuals with sALS has uncovered a notable increase in the methylation levels of genes related to calcium regulation (including those for calcium channels and a sodium-calcium exchanger), neurotransmission (genes involved in glutamate transport at synaptic vesicles), oxidative stress (genes related with repair of oxidative damage, and play a role in neuroinflammation) and mechanisms that are involved in protein aggregation (Morahan et al., 2009).

**Figure 1:**
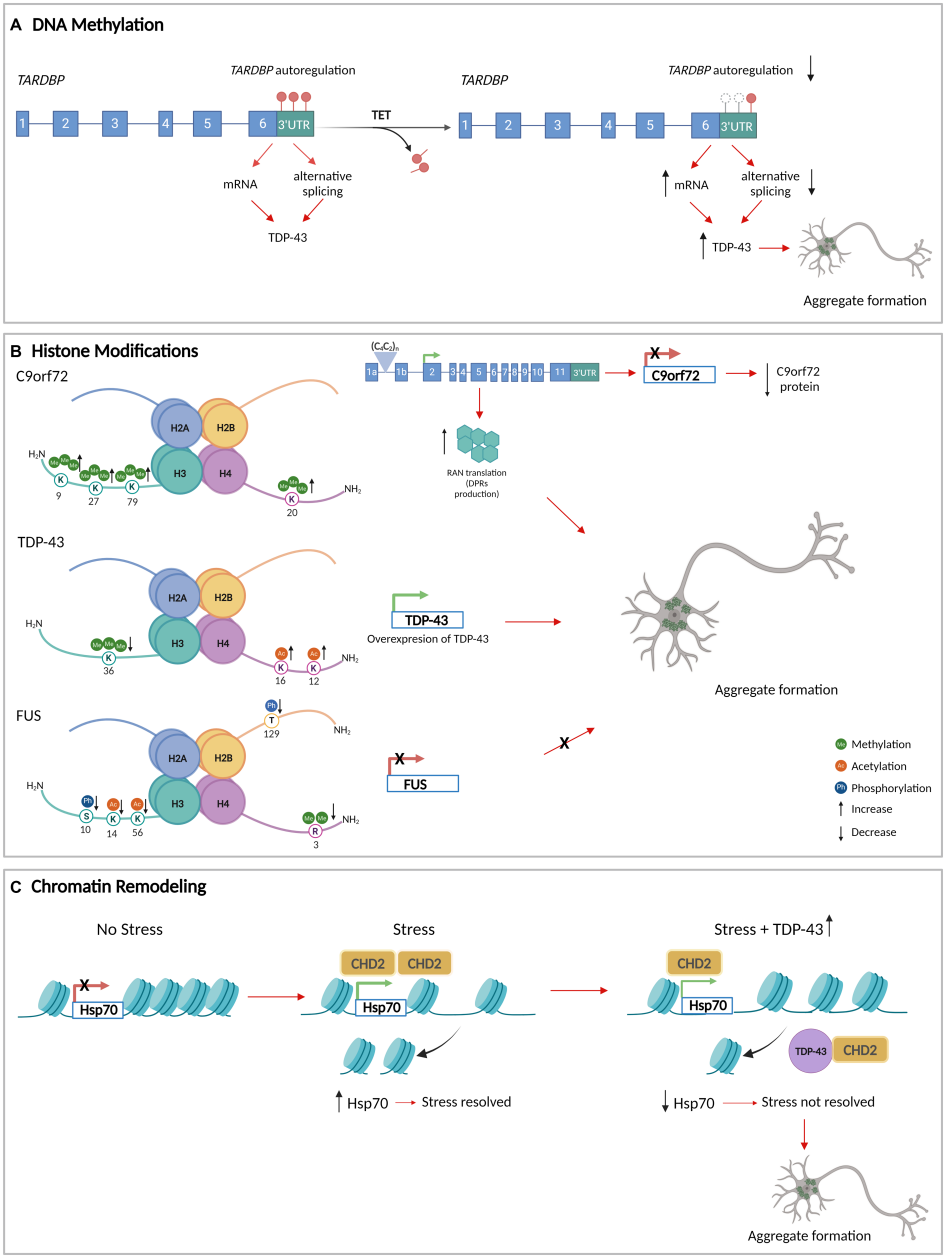
DNA methylation, histone modifications, and chromatin remodeling in aggregate formation in ALS. **(A)** The methylation of the DNA that encodes *TARDBP* 3’UTR (red circles) allows its autoregulation and normal levels of TDP-43. TET enzymes demethylate *TARDBP* 3’UTR DNA (white circles), decreasing the autoregulation and increasing the levels of TDP-43. **(B)** Histone modifications at the level of the genes that encode C9orf72, TDP-43, and FUS/TLS. The modifications generate an increase in the protein levels of TDP-43 with an increase in aggregate formation. For C9orf72 and FUS/TLS, histone modifications decrease the expression of the gene. In the case of C9orf72, the histones tri-methylation produces C9orf72 repeat expansions leading to repeat-associated non-AUG translation (RAN translation) resulting in dipeptide repeat proteins (DPRs) that accumulate as insoluble aggregates in the cytoplasm of neurons. **(C)** TDP-43 interacts with CHD2 impeding its recruitment in the chromatin. This results in the impairment of nucleosome clearance from the *Hsp70* gene which prevents its activation under stress. Consequently, there is an increase in TDP-43 levels and aggregation formation. Figure created with BioRender.com.

There is evidence that suggests the methylation status of the DNA is linked to the formation of pathological aggregates in ALS (Table 1) (Chen et al., 2012; Chouliaras et al., 2012). Reductions in methylated DNA in the presence of high expression levels of pathological TDP-43 have been described, suggesting a relationship between TDP-43 proteinopathy and DNA methylation (Appleby-Mallinder et al., 2021). Moreover, the DNA of the autoregulatory region that codifies the *TARDBP* 3’untranslated regions (3’UTR) is demethylated which is associated with reduced alternative splicing, increased levels of *TARDBP* mRNA, and in the end, augmenting the levels of cytoplasmic TDP-43 protein in affected neurons in ALS (Figure 1A) (Koike et al., 2021).

Methylation of the chromosome 9 open reading frame 72 (C9*orf*72) promoter region has been observed to decrease the gene expression, which may contribute to the reduction of RNA foci and dipeptide repeat aggregates formation (Babic Leko et al., 2019). This suggests that methylation of the C9*orf*72 promoter could have a neuroprotective role in ALS/FTD and conversely that demethylation could have a pathological effect.

Niclosamide, an anti-helminthic drug that reduces methylation in promoter CpG islands but increments methylation in the gene body region (Jiang et al., 2022) decreases the cytoplasmic accumulation of phosphorylated TDP-43 in the motoneurons of SOD1-G93A and FUS transgenic mice models. This drug also partially prevents the cytoplasmic mislocalization of FUS/TLS in the motoneurons (Milani et al., 2024). These results show that the hypomethylation in promoter CpG islands can decrease protein aggregation.

Other studies have examined site-specific promoter methylation and the expression of FUS/TLS and DNMTs in motor neurons derived from ALS patients with *FUS* mutations. Reduced *FUS* mRNA expression and cytoplasmic mislocalization of FUS/TLS leads to a loss of its nuclear function and the formation of toxic aggregates that represent a gain of function (Hartung et al., 2021). Both mechanisms may be partially driven by altered methylation. Higher expression levels of DNMT1, DNMT2, and DNMT3a have been observed in mutant *FUS* motoneurons (Hartung et al., 2021) as has been an inverse correlation of *FUS* expression and proximal *FUS* promoter methylation in ALS cell lines. Despite lower *FUS* expression levels, mutant *FUS* motor neurons show significantly more pathological cytoplasmic FUS/TLS aggregates suggesting that *FUS* methylation and repression act as protective mechanisms to counteract the formation of cytoplasmic aggregates (Hartung et al., 2021).

Finally, it has been observed that the methylation patterns from a specific region down to a single CpG can be identified using fragmentation patterns of circulating free DNA (cfDNA) molecules (Zhou et al., 2022b). A recent study found that methylation patterns on cfDNA from ALS patients accurately predicts ALS status and disease severity (Caggiano et al., 2024). Additionally, ALS cases were distinguished from FTD and other neurological conditions by the model. These findings suggest that cfDNA methylation patterns are potential quantitative biomarkers for ALS.

Nevertheless, while DNA methylation patterns may function as epigenetic indicators in ALS, it is still not clear if DNA methylation is the cause or consequence of protein aggregation and further research is necessary on this topic.

#### Histone modifications

Each nucleosome consists of DNA wrapped around a core of eight histone proteins, including two copies of each of histones H2A, H2B, H3, and H4 (Marino-Ramirez et al., 2005; Woodcock, 2005). The DNA wraps around this histone core forming the nucleosome structure together with histone H1 bound to the linker DNA. DNA segments connect adjacent nucleosomes aiding in the compaction of chromatin and the formation of higher-order chromatin structures (Widom, 1998). This organization of the chromatin helps to regulate gene expression, DNA replication, and DNA repair processes (Luger et al., 1997). The amino terminal tail of histones is exposed and subject to extensive PTMs such as acetylation (lysine residues), phosphorylation (serine or threonine residues), methylation (mono-, di-, and tri-methylation on lysine or arginine residues), SUMOylation (lysine residues), ubiquitination (lysine residues), glycosylation, and ADP-ribosylation (serine/threonine residues). These PTMs are important in gene regulation because they control the accessibility of DNA to the transcriptional machinery (both activation and inactivation) and chromatin remodeling processes without changing the DNA sequence (Becker and Workman, 2013; Stavreva and Hager, 2015; Allis and Jenuwein, 2016). Certain histone modifications, such as histone acetylation, are associated with active gene transcription, whereas methylations are associated with the formation of condensed and transcriptionally repressed chromatin (Heard et al., 2001; Bannister and Kouzarides, 2011).

The group of enzymes responsible for post-translational modification of histones includes histone acetyltransferase (HAT), histone methyltransferase (HMT), histone deacetylase (HDAC) and histone demethylase (HDM) (Hyun et al., 2017; Hwang et al., 2021; Liu et al., 2023a). Several of these enzymes are associated with ALS pathogenesis. For example, the reduction in the levels of cAMP-responsive element-binding protein (CREB)-binding protein (CBP) with HAT activity produces the histone H3 hypoacetylation detected in cholinergic motor neurons from the lumbar spinal cord in an ALS animal model (SOD1-G86R). This alteration has been associated with reduced motor neuron survival (Rouaux et al., 2003; Rouaux et al., 2007).

By acetylating histones H3 and H4, elongator complex protein 3 (ELP3) that has HAT activity directly controls the production of heat shock protein 70 (Hsp70) in yeast (Han et al., 2008). As a result, deficiencies in ELP3 may cause a reduction in Hsp70 transcription, which in turn may lead to motor neuron degeneration (Simpson et al., 2009). It has been shown that the knock-down of Hsp70 does not influence SGs assembly but results in the liquid-to-solid transition in SGs. Furthermore, by preventing the transition to a solid state, Hsp70 can assist in maintaining TDP-43-positive MLOs in a liquid state (Kitamura et al., 2018; Gu et al., 2021). Similarly, a propensity for liquid phase transition was observed with Hsp70 in FUS/TLS pathology. In this case, Hsp70 presence in FUS/TLS-related SGs correlates with a decrease in the transition from liquid to solid state (Li et al., 2022b).

In a variety of neurologic and psychiatric conditions, HDAC inhibitors can improve deficiencies in synaptic plasticity, cognition, and stress-related behaviors (Abel and Zukin, 2008). HDAC2 expression was found to be upregulated in the motor cortex and spinal cord grey matter, specifically in motor neuron nuclei of ALS patients, supporting the idea that the inhibition of HDAC2 has a protective role in the pathogenesis of ALS. The specific mechanism underlying this effect has not been described (Janssen et al., 2010). A selective increase in HDAC 2 levels has also been found in the muscle of a mouse model of muscular dystrophy, while functional and morphological parameters have been observed to improve with HDAC 2 downregulation in the same model (Minetti et al., 2006; Colussi et al., 2008). The neuroprotective role of HDAC reduction has also been observed using ACY-738, a strong HDAC inhibitor, in FUS/TLS transgenic mice. ACY-738 improves the motor phenotype and significantly increases the life span (Rossaert et al., 2019).

The link between histone modifications and aggregate formation has begun to be elucidated. In yeast, the deletion of a member of the HDAC complex known as a Set3 suppresses the toxicity of TDP-43 inclusions (Sanna et al., 2020). In SOD1 mutant cells, a connection between HDAC activity and aggresome formation has been observed through an intracellular structure that sequesters potentially toxic misfolded proteins and facilitates their clearance by autophagy. The percentage of cells containing aggresomes decreases when cells are treated with an HDAC inhibitor, implying a pathological role for HDACs (Corcoran et al., 2004). A study using a screening system for TDP-43 aggregation in mouse neuroblastoma Neuro2a cells analyzing a library of genes associated with fALS/FTD has shown that microtubule-related proteins (MRPs) and RBPs co-aggregate with TDP-43 in the cytosol via different mechanisms, involving microtubules and LLPS, respectively. TDP-43 aggregates induced by MRPs co-localize with markers for aggresomes and rely on HDAC6, further supporting a role for the aggresome formation in to aggregate formation (Watanabe et al., 2020).

The expression of HDAC4, an epigenetic factor responsive to stress, is significantly increased in the skeletal muscle of ALS patients and mice (SOD1-G93A) with denervation (Pigna et al., 2019) and is present in neuronal cytoplasmic inclusions (Federspiel et al., 2019). HDAC6 has been also implicated in protein aggregation through binding to ubiquitinated misfolded proteins (Simoes-Pires et al., 2013). Mutated SOD1 (mtSOD1) associated with ALS can regulate HDAC6 activity, leading to enhanced tubulin acetylation. This process subsequently promotes the aggregation of mtSOD1 in a process facilitated by dynein-mediated retrograde transport along microtubules (Gal et al., 2013).

Histone methylation has also been associated with ALS pathogenesis. Histone 3 (H3) trimethylation at lysine 9 (H3K9me3), 27 (H3K27me3), 79 (H3K79me3), and H4 trimethylation at lysine 20 (H4K20me3) have been shown to reduce C9*orf*72 gene expression in patients carrying pathological C9*orf*72 hexanucleotide repeat expansions (Belzil et al., 2013). These histone modifications create RNA foci that may sequester RBPs and serve as foci for C9*orf*72 dipeptide repeat protein aggregates formation (Ash et al., 2013; Mori et al., 2013) (Figure 1B). This suggests that the upregulation of H3 trimethylation-dependent C9*orf*72 expression could lead to dipeptide repeat protein aggregates formation.

It has been described that the bromodomain and extraterminal domain (BET) family of proteins that includes four conserved mammalian members of bromodomain-containing proteins (BRDs): BRD2, BRD3, BRD4, and BRDT, regulate the C9*orf*72 locus in ALS and that BRD inhibitors could have therapeutic potential for this disease (Zeier et al., 2015). These proteins function as epigenetic readers of histone acetylation, recruiting transcriptional regulator complexes to chromatin and binding to acetylated histones (Fujisawa and Filippakopoulos, 2017; Wang et al., 2021b; To et al., 2023).

BET inhibitors have been shown to effectively displace BET proteins from acetylated histones and elements of the transcriptional machinery (Filippakopoulos et al., 2010; Dawson et al., 2011; Delmore et al., 2011). Specifically, in primary cortical neurons from a C9*orf*72 ALS/FTD transgenic mouse model (C9BAC), treatment with PFI-1 and JQ1, both BRD inhibitors, enhanced the expression of the human mutant C9*orf*72 gene, increased the accumulation of nuclear RNA foci, and reduced poly (GP)-DPR inclusions, with reduced hippocampal-dependent cognitive impairments (Zeier et al., 2015).

Additionally, a role for a MAPK/MAK/MRK overlapping kinase (MOK), a Ser/Thr kinase in the mitogen-activated protein kinase (MAPK) superfamily, in regulating inflammatory and type-I interferon (IFN) responses in microglia through Brd4-dependent mechanisms has been uncovered (Perez-Cabello et al., 2023). MOK interacts with and colocalizes with cytoplasmic TDP-43 inclusions in microglia when these cells are exposed to external TDP-43 aggregates (Leal-Lasarte et al., 2017). It was found that MOK-mediated immune functions are dysregulated and actively contribute to ALS pathophysiology in a mouse preclinical model.

In yeast models, histone modifications and the expression of ALS-linked RBPs have been correlated. It has been observed that TDP-43^wt^ overexpression is associated with an increase in several histone PTMs, while FUS/TLS overexpression is associated with a decrease in histone modification levels. In the TDP-43 model, hyperacetylation of histone H4 on lysine 12 (H4K12ac) and 16 (H4K16ac) and reduced levels of trimethylation in histone H3 on lysine 36 (H3K36me3) have been observed, both of which result in increased *TARDBP* transcription (Figure 1B) (Chen et al., 2018). In the FUS/TLS yeast model, a decrease of dimethylation on arginine 3 of histone H4 (H4R3me2) has been observed as has been reduced levels of phosphorylation of H3 on serine 10 (H3S10ph) and H2B on threonine 129 (H2BT129ph), and hypoacetylation of H3 on lysine 14 (H3K14ac) and 56 (H3K56ac). These histone PTM changes collectively decrease *FUS* transcription (Chen et al., 2018) (Figure 1B). This suggests that hypermethylation of arginine 3, hyperphosphorylation of serine 10 and threonine 129, and hyperacetylation of lysine 14 and lysine 56 could be associated with an increase in FUS/TLS aggregate formation.

#### Chromatin remodeling enzymes

The chromatin remodeling complex is constituted by a group of proteins responsible for modulating chromatin architecture and is an essential component of the DNA damage response (Chou et al., 2010). This complex regulates gene expression temporally and spatially by altering transcriptional machinery accessibility and by controlling RNA polymerase-mediated transcription (Flaus et al., 2006; Wilson et al., 2021). The main families of chromatin remodelers include switch/sucrose non-fermenting (SWI/SNF), the imitation switch (ISWI), inositol requiring 80 (INO80), and the chromodomain helicase DNA binding proteins (CHD) (Clapier and Cairns, 2009).

The CHD protein family comprises nine DNA-binding proteins that contain chromo (chromatin organization modifier) and SWI/SNF helicase domains that are highly conserved between different organisms (Woodage et al., 1997). Beyond its role in regulating chromatin accessibility, CHD2 is also involved in DNA damage repair. CHD2 is recruited by poly (ADP-ribose) polymerase 1 (PARP1) and stimulates classical non-homologous DNA end joining (NHEJ), DNA repair (Al-Sarraj et al., 2011; Wang et al., 2013; Ray Chaudhuri and Nussenzweig, 2017). CHD2 triggers the deposition of histone variant H3.3 at sites of damage which facilitates the recruitment of X-ray repair cross-complementing protein 4 (XRCC4) and DNA ligase 4 (LIG4). The inhibition of NHEJ is associated with TDP-43 mislocalization in the cytoplasm, suggesting that persistent DNA damage and impaired DNA repair may further disrupt the nuclear function of TDP-43 in DNA repair, exacerbating nuclear DNA damage and the accumulation of TDP-43 in the cytoplasm (Al-Sarraj et al., 2011).

In normal conditions, CHD2, the homolog of CHD1 in *Drosophila melanogaster*, interacts with TDP-43 in modulating the stress response (heat shock and oxidative stress). Under these conditions, CHD2 facilitates nucleosome clearance from the Hsp70 transcription start site (TSS) of the gene, allowing its activation. However, through the interaction with CHD2, TDP-43 overexpression led to the inhibition of proper nucleosome clearance from the TSS of Hsp70, thereby impeding its activation and causing a failure in the stress response (Figure 1C) (Berson et al., 2017). In addition, the knockdown of CHD1 in *Drosophila melanogaster* induces SGs formation under oxidative stress, increasing both the number and size of SGs per cell and the percentage of cells with SGs (Berson et al., 2017). This supports the idea that CHD2 plays an important role in neuroprotection with the decrease of aggregate formation.

#### Non-coding RNAs

Non-coding RNA (ncRNAs) regulate gene expression at multiple levels in both the nucleus and cytosol using a variety of mechanisms. This property renders them critical elements in the fine control of the levels of RNAs and proteins involved in the formation of pathological aggregates. Certain long ncRNAs (lncRNAs) are also architectural in the formation of MLOs that have been involved in aggregate formation after stress. Any sustained alteration in this intricate network of thousands of ncRNAs and their targets that is not compensated for has the potential to lead to aberrant condensates that might evolve into insoluble aggregates through liquid-to-solid phase separation. This section is focused on those ncRNAs subgroups that regulate mRNA expression and levels of proteins of aggregates in ALS including examples of ncRNAs that control the function of these proteins and in doing so, are linked to granule and aggregate formation, maintenance, and removal.

##### microRNAs

Mature microRNAs (miRNAs) are 20–25 nt single stranded RNAs that regulate the expression of the whole human genome. In canonical biogenesis, miRNAs are formed from long primary sequences (pri-miRNAs) in sequential steps dependant on two ribonucleases, Drosha that processes pri-miRNAs in the nucleus, and Dicer that slices precursor miRNAs (pre-miRNAs) in the cytosol. MiRNAs bind through imperfect pairing with miRNA recognition elements (MREs) usually in 3’UTRs of RNA targets, which they use to guide Argonaute proteins from the RNA-induced silencing complex (RISC) to exert their slicing role and silence specific targets. Even though miRNAs are better known for their silencing effects at the transcript level, they can also activate or repress translation (Vasudevan et al., 2008; Treiber et al., 2019) and activate or repress transcription. The latter occurs through binding to complementary sequences in promoters and enhancers, with or without the participation of other epigenetic factors, interacting with single stranded DNA (miRNA: DNA) or double stranded DNA (miRNA:DNA:DNA) (Campos-Melo et al., 2022; Santovito and Weber, 2022; Hu et al., 2023).

MiRNAs are deeply interconnected with TDP-43 and FUS/TLS in that both RBPs have critical roles in miRNA biogenesis. TDP-43 regulates miRNA processing by interacting with Drosha and Dicer complexes and with selected pri- and pre-miRNAs (Kawahara and Mieda-Sato, 2012), while FUS/TLS facilitates miRNA processing by co-transcriptional recruitment of Drosha on the chromatin and binding to specific nascent pri-miRNA (Morlando et al., 2012). These functions have been linked to the vast downregulation of miRNAs observed in spinal cord in ALS, specifically in motor neurons (Campos-Melo et al., 2013; Emde et al., 2015). Further, the expression of pathogenic ALS-causing mutations of TDP-43 (A315T), FUS/TLS (R495X), and SOD1-G93A or their respective wild-type variants, or an oxidative stressor or endoplasmic reticulum (ER) stress was sufficient to reduce or inhibit Dicer catalytic activity (Emde et al., 2015).

**Figure 2:**
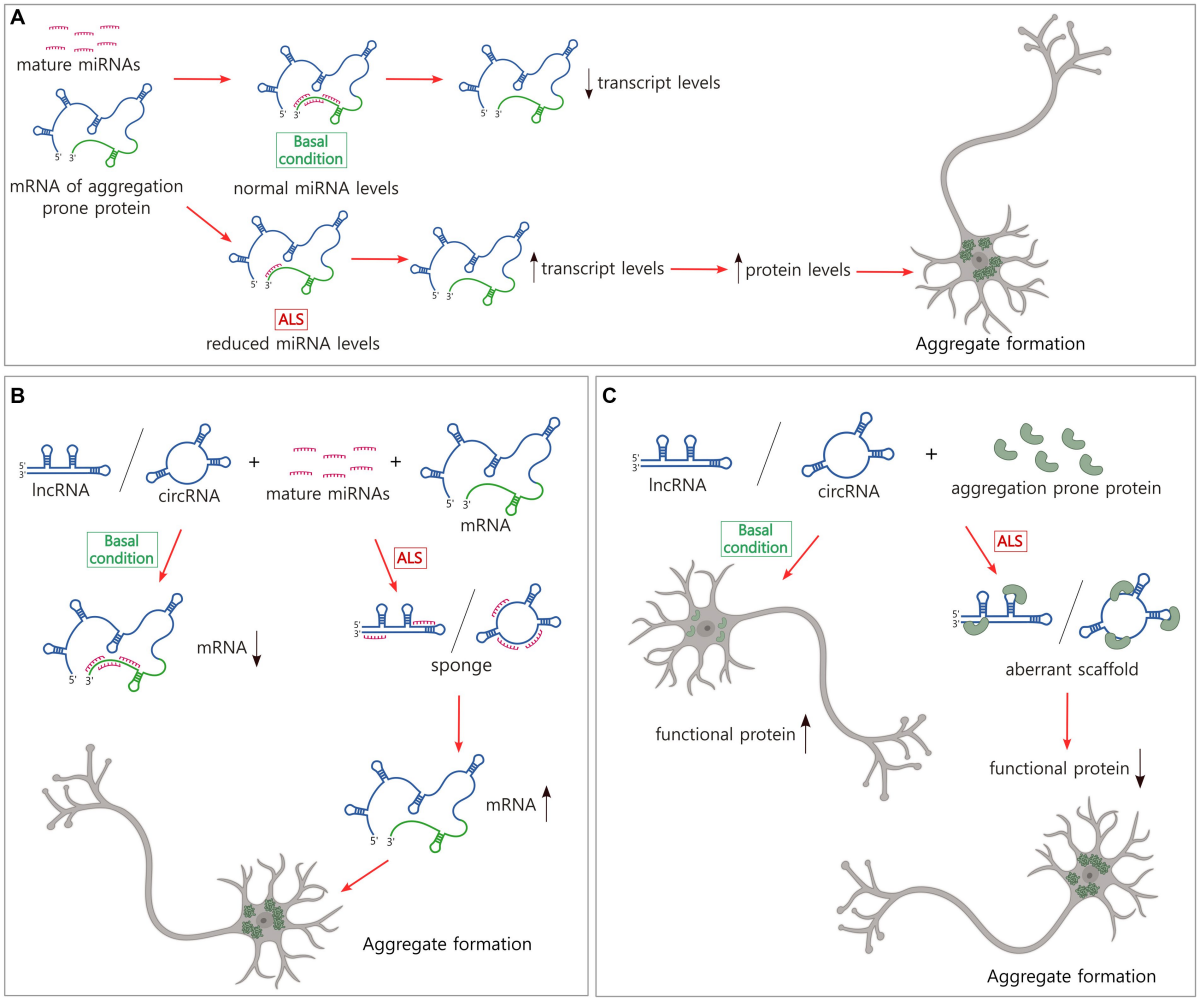
Non-coding RNAs modulate the levels of transcripts and proteins that form pathological inclusions in ALS. **(A)** The majority of miRNAs downregulate mRNA levels. In ALS, there is a broad reduction of miRNAs that might explain the increase in the levels of certain ALS-related transcripts and proteins and the subsequent aggregate formation. **(B)** LncRNAs and circRNAs sponge miRNAs which might increase the levels of certain transcripts and proteins, and the pathological aggregation. **(C)** LncRNAs and circRNAs bind to aggregation prone proteins, inducing aberrant scaffolds and reduction of levels of functional proteins. This loss of balance could generate protein inclusion formation. Figure created with BioRender.com.

Numerous miRNAs have been reported to modulate the levels of TDP-43, FUS/TLS, and other proteins involved in aggregate formation in ALS or the regulation of this process (Liu et al., 2022a) (Table 1). Many of these miRNAs have been studied in detail in the context of cancer; however, examples of miRNAs that are linked to neurodegeneration, and specifically to ALS, have also been reported (Figure 2A). For instance, miR-27b-3p and miR-181c-5p, two miRNAs that are reduced in ALS, downregulate TDP-43 expression as a part of a negative feedback loop dependent on TDP-43 nuclear localization (Campos-Melo et al., 2013; Hawley et al., 2020). It is important to note that miRNAs have multiple targets. Thus, a single miRNA can alter the levels of different proteins associated with aggregate formation leading to complex effects. This is the case of miR-183-5p, which has been reported to be upregulated in the spinal cord in ALS and downregulated in the hippocampus and cerebellum (Kim et al., 2023). MiR-183-5p is induced after stress and increases neuronal survival by directly targeting programmed cell death 4 (*PDCD4*) and receptor interacting serine/threonine kinase 3 (*RIPK3*) transcripts (Li et al., 2020). In addition, miR-183-5p reduces sequestosome-1/ubiquitin-binding protein p62 (SQSTM1/p62) and increases TDP-43 levels in neuronal cells, regulating the formation of SGs and protecting against cytotoxicity (Kim et al., 2023). Other examples of miRNAs with several ALS-linked targets are miR-194 and miR-b2122 in which the former is involved in the regulation of inflammation and the latter a novel miRNA. Both miRNAs regulate TDP-43 and FUS/TLS and show reduced levels in ALS patients (Hawley et al., 2017).

One of the most studied miRNAs is miR-206, which is upregulated in blood samples of ALS patients (Toivonen et al., 2014; Dobrowolny et al., 2021; Gomes et al., 2023). MiR-206 is also significantly upregulated in the skeletal muscle of SOD1-G93A transgenic mice in which motor deficits are expressed. MiR-206 ^−/−^; SOD1-G93A mice showed that loss of miR-206 accelerated the progression of motor impairments and decreased survival (Williams et al., 2009). Details of the mechanism include that miR-206 downregulated the translation of HDAC4 *in vitro* and both have opposite effects on fibroblast growth factor binding protein 1 pathway, promoting and impeding neuromuscular junction innervation after injury, respectively (Williams et al., 2009). This study and the protective role of HDAC inhibitors in animal models (described in the histone modifications section) support the idea that increased levels of miR-206 in the skeletal muscle might have a protective function in ALS. However, an investigation that analyzed muscle samples of a small group of ALS patients observed that HDAC4 levels are increased and occurred exclusively in rapidly progressing ALS, and that only HDAC4 (not miR-206) had a correlation with reinnervation (negative) and disease progression rate (positive) (Bruneteau et al., 2013).

Other studies have explored the link between miR-206 and aggregate formation in neurodegeneration. It has been reported that miR-206 downregulates Fas apoptotic inhibitory molecule (FAIM), a protein implicated in blocking the formation of protein aggregates under stress conditions and whose isoforms are de-regulated in Alzheimer’s disease and dopaminergic neurons (Yu et al., 2008; Carriba et al., 2015; Coccia et al., 2020). In *in vitro* experiments, FAIM counteracted SOD1-G93A aggregation in cells in filter trap and sedimentation assays (Kaku et al., 2020). Altogether these results suggest that the upregulation of miR-206 and the reduction of HDAC4 might be protective in SOD1-G93A mice. However, in patients, there could be elements in this regulatory network that partially block the beneficial effect of miR-206. It would be interesting to study specific regulatory molecules of miR-206, such as the hepatocyte growth factor and circular_0057558 (circ_0057558) (Choi et al., 2018; Chen et al., 2021b), as well as investigate FAIM levels in ALS patients. It is possible that the upregulation of miR-206 might be increasing the formation of aggregates via FAIM reduction. Finally, the multilayer mechanism of action of miR-206 also included the interaction of miRNA-206 with TDP-43, which limits miRNA activity by disrupting their RISC association (King et al., 2014).

##### Long non-coding RNA and circular RNA

LncRNAs are molecules of ≥200 nt in length that show diverse mechanisms of biogenesis. Many lncRNAs are spliced, 7-methylguanosine capped, and polyadenylated as mRNAs; others are not. LncRNAs are expressed from RNA polymerase I (Pol I) or Pol III promoters or are processed from precursors that come from introns or repetitive elements. LncRNAs can also be antisense with respect to protein coding genes, transcribed from intergenic regions or pseudogenes, or derived from 3’UTR mRNAs (Mercer et al., 2011; Mattick et al., 2023). LncRNAs can be linear or circular (circRNAs), and the majority of the latest generated by back-splicing of coding and non-coding transcripts using the spliceosomal machinery (Liu and Chen, 2022).

Linear lncRNAs (called lncRNAs hereafter) and circRNAs have multiple dynamic functions in the regulation of gene expression but only a limited group have been characterized in neurodegeneration. LncRNAs act as guides and scaffolds, binding transcription factors and chromatin-modifiers, and assembling protein complexes to direct them to specific genomic locations. They can function as molecular decoys, sponging miRNAs inhibiting their binding to target mRNAs or sequestering regulatory proteins and transcription factors, and chromatin modifiers from gene promoters. LncRNAs also have enhancer-like functions, activating gene transcription in *cis* and circRNAs regulate splicing of protein coding transcripts (Kopp and Mendell, 2018; Liu and Chen, 2022; Mattick et al., 2023). While studies of the regulatory mechanisms utilized by lncRNAs and circRNAs are also abundant in the cancer field, our understanding of their role in ALS is still early. However, given that the functional strategies these molecules use are extensively spread in nature, further examining their role in aggregate formation in ALS is timely (Figures 2B,C).

Currently, a limited number of lncRNA expression profiles exist for ALS tissue. The most representative examples show the dysregulation of this population of ncRNAs in the blood cells of ALS patients (Gagliardi et al., 2018; Yu et al., 2022). Among the lncRNAs that modulate the levels of expression of ALS-linked genes (Table 1) is SOD1-DT, a divergent lncRNA encoded near the locus of *SOD1* gene that is transcribed in the opposite direction. SOD1-DT is expressed in neuronal cells and regulates the expression of *SOD1* using an unknown mechanism, suggesting a role in ALS (Guerra et al., 2023). Other examples are long intergenic non-protein coding RNA 2231 (linc02231) and lncRNA ANCR that sponge miR-939-5p and miR-140-3p, respectively, and prevent heterogeneous nuclear ribonucleoprotein A1 (hnRNPA1) degradation (Wen et al., 2020; Xu et al., 2023), and lncRNA SOX2-OT that sponges miR-146b-5p increasing the levels of hnRNPA2B1 (Zhang and Li, 2019). In the pathogenesis of ALS, it has been observed that lnc-HIBADH-4 levels correlate with disease severity and survival. Lnc-HIBADH-4 is another example of a molecular sponge, but it controls the clearance of protein aggregates in neurons by regulating lysosomal function through the lnc-HIBADH-4/miR-326/cathepsin D pathway (Figure 2B) (Huang et al., 2023).

Nuclear paraspeckle assembly transcript 1 (NEAT1) is a lncRNA that functions as a scaffold of proteins in the formation of nuclear paraspeckles, MLOs that modulate gene expression by sequestering mRNAs from translation and proteins from their gene regulatory functions (Hirose et al., 2014; Jacq et al., 2021; McCluggage and Fox, 2021). At a physiological level, paraspeckles protect the cell in stress conditions and have been involved in different neurodegenerative diseases (An et al., 2018). TDP-43, FUS/TLS, TATA-box binding protein associated factor 15 (TAF15), Ewing’s sarcoma RBP 1 (EWSR1), and HNRNPA1, all proteins that form pathological aggregates in ALS, are recruited in paraspeckles (Naganuma et al., 2012). Then, alterations in NEAT1 levels compromise the abundance and biophysical properties of paraspeckles, the availability of free paraspeckle proteins, and as with other MLOs, might potentially induce the formation of protein aggregates in neurodegeneration (Figure 2C). In fact, it has been observed that in the early phase of ALS, NEAT1_2, one of the two isoforms of NEAT1, is upregulated, binds to TDP-43 and FUS/TLS, and induces paraspeckle formation in motor neurons, modulating the levels and functions of these ALS-associated RBPs (Nishimoto et al., 2013). Reduction of nuclear TDP-43 but not its accumulation in the cytosol or aggregation results in this paraspeckle hyper-assembly (Shelkovnikova et al., 2018). Poly-proline-arginine, the most toxic dipeptide repeat from C9*orf*72, binds to and upregulates NEAT1, increasing also paraspeckle formation (Suzuki et al., 2019).

The expression and function of circRNAs in ALS is also beginning to be elucidated. Two independent groups have analyzed the levels of circRNAs in skeletal muscle and spinal cord in ALS patients to evaluate their biomarker potential. It has been observed that a group of circRNAs is altered in ALS-relevant tissues, some of them in opposite directions (Aquilina-Reid et al., 2022; Tsitsipatis et al., 2022). Evidence that might link circRNAs with aggregate formation in ALS arises mainly from FUS/TLS studies. FUS/TLS and circRNAs are closely interrelated. FUS/TLS regulates circRNA biogenesis by binding the introns flanking the back-splicing junctions inducing cyclization (Errichelli et al., 2017; He et al., 2023; Liu et al., 2023b). However, circRNAs such as circRNA-0004904 and circRNA_0000285 also modulate FUS/TLS levels (Chen et al., 2019; Dai and Liu, 2021). In a different mechanism, circRNAs recruit/sponge FUS/TLS to participate in mRNA stability in different regulatory axes that involve SGs (Liu et al., 2023b; Liu et al., 2023c).

The intracellular traffic of the multi-exonic circ-Hdgfrp3 has also provided some insights. In wild-type stressed motor neurons, circ-Hdgfrp3 localized to SGs are proposed to serve as a protein scaffold. In motor neurons carrying mutant FUS/TLS, a high proportion of circ-Hdgfrp3 is trapped in cytoplasmic aggregates, from where it’s not efficiently released upon stress removal (D'Ambra et al., 2021). Aggregation of TDP-43 might also be connected to circRNAs function. The overexpression of circTmeff1 triggers skeletal muscle atrophy *in vivo* and *in vitro*, and its knockdown partially rescues muscle mass in an atrophy model in mice (Chen et al., 2023). Mechanistically, circTmeff1 directly interacts with TDP-43 and promotes its aggregation in mitochondria (Figure 2C), triggering the release of mitochondrial DNA into the cytosol and the activation of the cyclic GMP-AMP synthase (cGAS)/stimulator of interferon genes (STING) pathway (Chen et al., 2023).

### RNA modifications

The epitranscriptome describes changes to the transcriptome that do not involve changes in the ribonucleotide sequence but are based on nucleotide chemical modifications (Saletore et al., 2012). There are more than 100 different types of chemical modifications identified for RNA in both coding RNAs and noncoding RNAs during their cycle of life (Roundtree et al., 2017) including N6-methyladenosine (m6A) (Meyer et al., 2012), N1-methyladenosine (m1A) (Safra et al., 2017), and inosine-to-adenine editing (Bazak et al., 2014). Some of them are relevant to ALS.

#### RNA methylation

m6A is the most abundant conserved modification and plays a crucial role in regulating gene expression. This modification has been extensively associated with nuclear splicing, mRNA stability, translation speed, and the subcellular localization of targeted mRNAs (Widagdo and Anggono, 2018). For lncRNAs, m6A regulates splicing and nuclear export (Akhtar et al., 2021). m6A is catalyzed by the Mettl3/Mettl14 methyltransferase complex and can also undergo demethylation by fat mass and obesity-associated protein (FTO) and AlKBH5 (AlkB family homolog 5) demethylases. m6A are recognized by “readers” such as the YT521-B homology (YTH) domain family proteins (YTHDFs), YTH domain-containing proteins (YTHDCs), insulin-like growth factor 2 mRNA-binding proteins (IGF2BPs), and eukaryotic initiation factor 3 (eIF3), potentially influencing mRNA translation, stability, splicing, or localization (Scuteri et al., 2007; Jia et al., 2011; Wang et al., 2016a).

It has been shown that TDP-43 interacts with its own m6A-modified RNA and in doing so downregulates its expression. The extensive RNA hypermethylation in post-mortem spinal cord tissue from sALS patients also suggests a direct association between the regulation of TDP-43 levels in ALS and m6A modification (McMillan et al., 2023). Moreover, an overlap between m6A and cytoplasmic TDP-43 inclusions has been observed in motor neurons (McMillan et al., 2023), and recently, a mechanism by which m1A CAG repeat RNA binds TDP-43 and stimulates its mislocalization in the cytosol and the formation of gel-like aggregates has been described (Sun et al., 2023). Together these studies confirm TDP-43’s capacity to detect methylated RNA and the link between this modification and protein aggregate formation in neurodegeneration.

#### RNA editing

RNA editing involves post-transcriptional modifications mediated by cytosine and adenosine deaminases, wherein nucleotides are modified, inserted, or deleted, often facilitated by enzymes, sometimes modifying the RNA’s coding potential. Apobec enzyme naturally converts cytosines to uracils, while adenosine deaminases, such as the adenosine deaminases acting on RNA (ADARs) enzyme family, transform adenines into inosines (Tan et al., 2017; Walkley and Li, 2017). These changes can influence RNA translation into proteins, potentially impacting protein folding and aggregation. Furthermore, RNA editing can influence RNA stability and localization, affecting protein aggregation dynamics (Eisenberg and Levanon, 2018).

In sALS motor neurons, there is a concomitant occurrence of TDP-43 aggregation and reduced expression of ADAR2 (Aizawa et al., 2010; Hideyama et al., 2012). The enzyme ADAR2 primarily targets the Q/R site in GluA2 (a subunit of AMPA receptor) pre-mRNA and its deficiency leads to excessive Ca^2+^ permeability of glutamate-AMPA receptors due to the failure of A-to-I editing (Kawahara et al., 2004; Kawahara and Kwak, 2005). This conversion is crucial for motor neuron survival (Kwak and Kawahara, 2005; Hideyama et al., 2010). The loss of ADAR2 promotes the activation of calpain proteases due to this excess of Ca^2+^ permeability, which induces cleavage of the TDP-43 C-terminal fragment, generating aggregation-prone N-terminal fragments. Persistent calpain activation results in the gradual expansion of TDP-43 aggregates, contributing to disease progression (Yamashita and Kwak, 2014). The evidence shows that the variation in TDP-43 expression does not affect the expression levels of ADAR2, suggesting that the aggregation or abnormal TDP-43 processing is a consequence of an inefficient GluA2 Q/R site RNA editing in the motor neurons of sALS patients (Yamashita et al., 2012).

Notably, a deficiency of ADAR2 was also observed in the spinal motor neurons of an ALS patient with a FUS/TLS mutation, although it is still unclear if this deficiency is related to the presence of FUS-positive inclusions (Aizawa et al., 2016). In addition, ADAR2 undergoes nucleocytoplasmic mislocalization, leading to abnormal RNA editing in the postmortem tissue of individuals with C9*orf*72 mutations. A significant proportion of neurons with cytoplasmic accumulation of ADAR2 along with TDP-43 pathology was observed in the spinal cord of C9*orf*72 ALS/FTD patients (Moore et al., 2019). In this study, the analysis of the whole transcriptome for RNA A-to-I editing changes was evaluated by RNA-seq, indicating that these RNA alterations occur in 1,526 genes, including ALS-related transcripts (Moore et al., 2019).

### Aging and epigenetic modifications

Currently, the evidence suggests that the formation of protein aggregates and epigenetic modifications can also be related to aging (Lopez-Otin et al., 2023). The alterations of epigenetic modifications affect DNA replication and repair, gene transcription and silencing, cell division, and telomere length maintenance (Gonzalo, 2010). With age, chromatin undergoes various changes, such as structural remodeling, alterations in chromatin architecture, loss of histones, and modifications to histones. For example, reduced global histone acetylation can disrupt metabolic gene expression and homeostasis. The balance of histone acetylation and deacetylation is critical, and any disruption in this balance can lead to issues in stress response and DNA repair mechanisms (Eberharter and Becker, 2002; Lazo-Gomez et al., 2013; Jagaraj et al., 2024).

In the context of ALS pathology, it has been described that aging accelerates DNA methylation in the CpG-island 5′ region which is linked to a more severe ALS disease phenotype, earlier onset, and shorter disease duration in patients with C9*orf*72 mutations (Zhang et al., 2017).

It makes sense to hypothesize that alterations of the epigenome due to aging could trigger or contribute to a cascade of events that could contribute to the formation of protein aggregates in pathologies such as ALS. However, this is a vastly unexplored field, and more studies are necessary to elucidate this link.

### Final remarks

In this review, we presented the current evidence that suggests how epigenetic mechanisms can be related to inclusion formation in ALS, and by extension to other related neurodegenerative diseases. This holds significant therapeutic potential for ALS since targeting specific epigenetic regulators involved in protein aggregation pathways could offer novel strategies for mitigating neuronal dysfunction and slowing disease progression in ALS patients. In the course of studying the evidence of the link between epigenetics and inclusion formation, we realized two main hindrances in the field: first, the definition of epigenetics itself, and second, the causality association between epigenetics and biomolecular condensate formation.

The definition of epigenetics has been a controversial topic since it was first conceptualized (Felsenfeld, 2014). Many definitions have been created since then, depending especially on the field to which the author belongs (Nicoglou and Merlin, 2017). In general, the contemporary definition includes DNA modifications, chromatin regulation, and the possibility of heritability. However, where the definition becomes open to interpretation is concerning the role of RNA in epigenetics. In this work, we used the contemporary definition of Cavalli (Cavalli and Heard, 2019) which includes RNA (ncRNA and RNA modifications). However, it is clear to us that this boundary is diffuse. The RNA field has had an explosive development over the last decade and the question that emerges is if it will be necessary to include all the RNA-related regulatory processes as a separate mechanism with its own definition, leaving only the chromatin-related mechanisms to the epigenetics field.

Despite the evidence showing how epigenetics can affect directly or indirectly the aggregate formation in ALS, we also noticed several examples where only a correlation between both was detected. It becomes quite complex trying to elucidate which one has a causal role. Specifically, it remains unclear in many cases if changes in epigenetics modifications are leading to disease processes or if disease processes lead to changes in the epigenome. In this context, the field seems rather stalled with few mechanistic studies published. We believe that greater efforts are needed in deciphering these causal processes.

As the majority of ALS cases do not have a clear genetic explanation, studying the epigenome becomes highly relevant for understanding the pathogenesis of ALS and the goal of finding new therapeutic targets. This would also help us to understand if feedback loops between aggregate formation and epigenetics alterations exist, a key point in the design of new drugs that could reduce or stop the disease progression.
